# Spatially resolved transcriptomics and graph-based deep learning improve accuracy of routine CNS tumor diagnostics

**DOI:** 10.1038/s43018-024-00904-z

**Published:** 2025-01-29

**Authors:** Michael Ritter, Christina Blume, Yiheng Tang, Areeba Patel, Bhuvic Patel, Natalie Berghaus, Jasim Kada Benotmane, Jan Kueckelhaus, Yahaya Yabo, Junyi Zhang, Elena Grabis, Giulia Villa, David Niklas Zimmer, Amir Khriesh, Philipp Sievers, Zaira Seferbekova, Felix Hinz, Vidhya M. Ravi, Marcel Seiz-Rosenhagen, Miriam Ratliff, Christel Herold-Mende, Oliver Schnell, Juergen Beck, Wolfgang Wick, Andreas von Deimling, Moritz Gerstung, Dieter Henrik Heiland, Felix Sahm

**Affiliations:** 1 Dept. of Neuropathology, University Hospital Heidelberg, Heidelberg, Germany; 2 Clinical Cooperation Unit Neurooncology, German Consortium for Translational Cancer Research (DKTK), German Cancer Research Center (DKFZ), Heidelberg, Germany; 3 Department of Neurological Surgery, Washington University in St. Louis School of Medicine, St. Louis, MI USA; 4 Department of Neurological Surgery, Northwestern University Feinberg School of Medicine, Chicago, IL USA; 5 Department of Neurosurgery, University Hospital Erlangen, Erlangen, Germany; 6 Microenvironment and Immunology Research Laboratory, Friedrich-Alexander Universität Nürnberg-Erlangen, Erlangen, Germany; 7 Translational Neurosurgery, Friedrich-Alexander Universität Nürnberg-Erlangen, Erlangen, Germany; 8 Department of Neurosurgery, Medical Center - University of Freiburg, Freiburg, Germany; 9 Faculty of Medicine, Freiburg University, Freiburg, Germany; 10 Division of AI in Oncology, German Cancer Research Center (DKFZ), Heidelberg, Germany; 11 Department of Neurosurgery, Hospital Memmingen, Memmingen, Germany; 12 Department of Neurosurgery, University Medicine Mannheim, University of Heidelberg, Mannheim, Germany; 13 Division of Experimental Neurosurgery, University Hospital Heidelberg, Heidelberg, Germany; 14 Division of Pediatric Neurooncology, German Consortium for Translational Cancer Research (DKTK), German Cancer Research Center (DKFZ), Heidelberg, Germany; 15 National Center for Tumor Diseases (NCT), Heidelberg, Germany; 16 Department of Neurology, University Hospital Heidelberg, Heidelberg, Germany; 17 German Cancer Consortium (DKTK), Partner Site Freiburg, Freiburg, Germany

**Keywords:** Diagnostic markers, Cancer, CNS cancer, Machine learning

## Abstract

The diagnostic landscape of brain tumors integrates comprehensive molecular markers alongside traditional histopathological evaluation. DNA methylation and next-generation sequencing (NGS) have become a cornerstone in central nervous system (CNS) tumor classification. A limiting requirement for NGS and methylation profiling is sufficient DNA quality and quantity, which restrict its feasibility. Here we demonstrate NePSTA (neuropathology spatial transcriptomic analysis) for comprehensive morphological and molecular neuropathological diagnostics from single 5-µm tissue sections. NePSTA uses spatial transcriptomics with graph neural networks for automated histological and molecular evaluations. Trained and evaluated across 130 participants with CNS malignancies and healthy donors across four medical centers, NePSTA predicts tissue histology and methylation-based subclasses with high accuracy. We demonstrate the ability to reconstruct immunohistochemistry and genotype profiling on tissue with minimal requirements, inadequate for conventional molecular diagnostics, demonstrating the potential to enhance tumor subtype identification with implications for fast and precise diagnostic workup.

## Abstract (web summary)

Ritter et al. present a spatial transcriptomics and deep learning-based approach named NePSTA (neuropathology spatial transcriptomic analysis) and leverage it to improve neuropathological diagnostics and enhance central nervous system tumor subtype classification.

## Main

Recent advances in brain tumor classification have transformed neuropathological diagnostics in daily routine. Traditional diagnostic features, such as morphological appearance and single-protein expression, are now complemented or even replaced with complex molecular markers assessed by next-generation sequencing (NGS) and DNA methylation analysis^1,2^. Genome-wide DNA methylation profiling has dramatically reshaped brain tumor classification and was, hence, incorporated into the 2021 World Health Organization (WHO) classification of central nervous system (CNS) tumors^2–5^. This comprehensive methylation profiling not only improves differentiation of tumor types, with substantially higher accuracy in identifying rare tumor variants, but also reveals novel tumor subtypes^4^. However, the granularity of molecular diagnostics presents technical challenges, particularly the requirement for high-quality, abundant DNA, which in turn requires sufficient tissue^6^. This becomes especially pivotal for highly eloquent tumors where small biopsy samples often preclude extensive morphological workup with serial immunohistochemistry (IHC) and yield limited DNA, preventing comprehensive molecular diagnostic evaluation. In addition, samples with a very low tumor content might also prevent a complete molecular workup for diagnosis. Hence, information crucial for subsequent management and therapeutic targeting remains elusive. Spatially resolved transcriptomics, previously applied mainly to explore spatial architecture in brain tumors, offers a solution^7,8^. Using paraffin-embedded sections, the Visium (10X) technology requires just a single 5–10-µm section comparable to a single regular IHC stain yet delivers robust expression profiles with spatial precision. Its ability to generate high-dimensional data from minimal tissue holds potential as an invaluable addition to the neuropathological diagnostic arsenal, especially for challenging tissues.

In this study, we developed and validated a diagnostic application of spatially resolved transcriptomics, offering a computational framework, named NePSTA (neuropathology spatial transcriptomic analysis), based on classical machine learning algorithms and graph-based artificial intelligence (AI) application for neuropathological diagnostics. This framework encompasses the entirety of contemporary molecular-based neuropathological diagnostics including automated histological evaluations, transcriptomics and genetic and epigenetic profiling. Assessing 130 participants across a spectrum of neuroepithelial tumors and healthy donors acquired from four distinct medical centers, we integrated expression levels and inferred copy-number alterations (CNA) to train a graph neural network (GNN) to predict histological contexts and methylation-based subclasses. The result was a striking accuracy rate of 89.3% on a participant level.

## Results

### Overview of the training and test cohorts

**Fig. 1:**
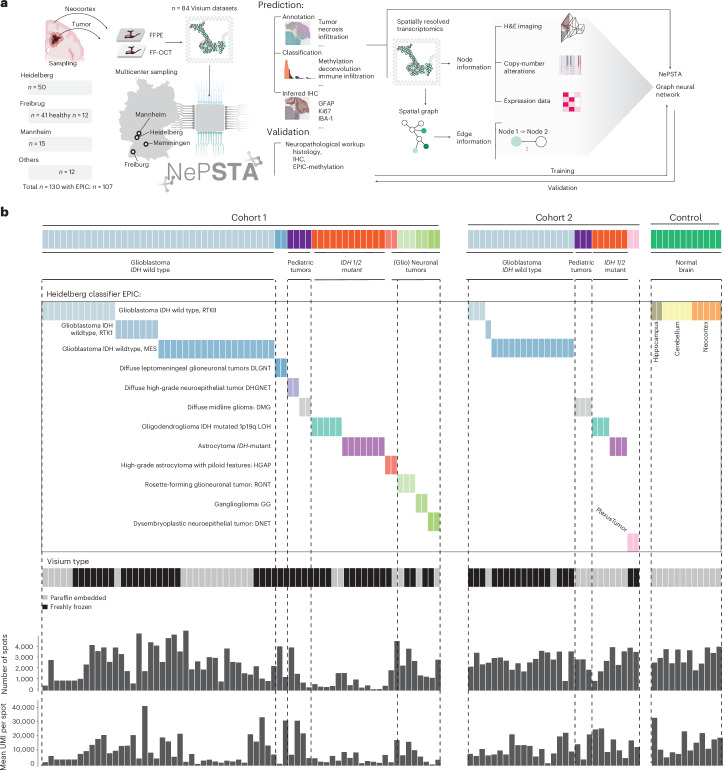
Overview of the cohort. **a**, Illustration of the workflow and computational pipeline. **b**, Top: overview of the cohorts (*n* = 107 participants) with histological diagnosis. Middle: methylation-based classification. Colors indicate the type of methylation-based subgroup derived from the Heidelberg classifier. Bottom: tissue type characteristics and quality control data are demonstrated. Top: bar graph showing the total number of spots per participant. Bottom: bar graph illustrating the total number of UMIs per participant.

Leveraging spatially resolved transcriptomics through the Visium technology, we achieved robust mRNA profiling from minimal 5-µm paraffin-embedded tissue sections. This method’s capability to derive extensive molecular insights from small biopsy fragments—where traditional DNA extraction often fails or tumor cell fraction is low—guided our hypothesis. We posited that integrating expression data with their spatial context could predict genomic features such as copy-number variations (CNVs), provide molecular diagnoses (Heidelberg classifier) and enable inferred immunostainings from a single tissue slice (Fig. 1a). Our study involved samples from four German medical centers (Heidelberg, *n* = 50; Freiburg, *n* = 41; Mannheim, *n* = 15; Memmingen, *n* = 1), along with external controls published recently^9^ (*n* = 11) and 12 healthy cortex samples, totaling 130 samples. These were divided into two cohorts. The first cohort (*n* = 66) comprised samples from three centers, encompassing a range of CNS pathologies from highly malignant glioblastomas (GBs) to epilepsy-associated (glio)neuronal tumors. These samples underwent comprehensive spatial transcriptomics profiling, alongside state-of-the-art diagnostic workups, including morphology inspection, EPIC methylation arrays, classifier predictions, IHC and NGS. The second cohort (*n* = 41) was a single-center cohort from Freiburg, similarly profiled (Fig. 1b). We hypothesized that the composition of the tumor and neural environment form a distinct molecular fingerprint for each CNS pathology, which can be captured using spatially resolved transcriptomics. Our aim was to leverage these fingerprints to predict epigenetically determined tumor subclasses as defined by the Heidelberg classifier. Additionally, the versatility of spatial transcriptomics technology enabled several objectives: authenticating traditional neuropathological diagnostics, comparing the efficacy of spatial transcriptomics against molecular workups or classical morphological evaluations and enhancing precision in distinguishing normal from pathological tissues by analyzing control tissues (neocortex, *n* = 5; cerebellum, *n* = 5; hippocampus, *n* = 2). We also aimed to extend molecular profiling to sparse samples previously unsuitable for comprehensive analysis.

### Robust quality and readouts across spatial gene expression technologies and institutions

First, we aimed to explore and quantify potential confounders of the technology, as clinical applications demand robust and consistent quality readouts. In our cohort, samples were processed either at the Department of Neuropathology in Heidelberg (cohort 1) or at the Neurosurgical Department in Freiburg (cohort 2). In cohort 1, the majority of samples (*n* = 42, 63.6%) were processed from paraffin-embedded tissue, with only 24 samples (36.6%) processed from freshly frozen tissue. Conversely, cohort 2 included approximately half of the samples (*n* = 18, 43.9%) processed from paraffin-embedded tissue. Leveraging the mean unique molecular identifier (UMI) per spot, we did not detect significant differences between the cohorts or sample types in terms of overall quality metrics (Fig. 1b). However, upon closer examination, we found that certain biological and technical factors contribute to the variation in UMI counts across different tissue types and sample preparation methods. Both cohorts demonstrated a strong correlation between cellular density and the number of UMIs per spot. *IDH-*wild-type GB samples consistently exhibited the highest number of UMIs per spot because of their increased cellular density and RNA abundance. The variance in the number of spots under tissue was largely influenced by the source of the tissue, whether it was a biopsy (smaller tissue fragment) or a bulk resection (larger tissue fragment). Biopsy samples, particularly in cohort 1, were more common in lower-grade tumors such as astrocytomas, leading to fewer spots overall because of the smaller tissue area sampled. The lower spot count in cohort 1 is, thus, a reflection of sample size and type rather than an indicator of data quality. In nonmalignant tissue samples, the number of cells per spot ranged from 0 to 5 cells per spot, which is notably lower compared to GB samples, where the cellular density was higher, ranging from 7 to 15 cells per spot. This discrepancy in cellular density is mirrored by the number of UMIs detected per spot, with lower UMI counts consistently observed in healthy brain samples (Fig. 1b). The lower UMI counts in nonmalignant tissues and lower-grade gliomas, such as astrocytomas, can be attributed to several factors. First, the cellular composition of nonmalignant and lower-grade tumors differs greatly from that of high-grade tumors. For example, nonmalignant regions tend to contain more myeloid cells, which have lower RNA content, resulting in fewer UMIs. In contrast, regions with higher neuronal content—such as those in GB samples—have more abundant RNA, contributing to higher UMI counts^10^. Therefore, the variation in UMI counts between samples reflects both the cellular density and the composition of the tumor microenvironment. Additionally, technical factors such as sequencing depth and sample preparation methods can impact UMI counts. For instance, freshly frozen samples generally yield higher-quality RNA and more UMIs than formalin-fixed paraffin-embedded (FFPE) samples, where RNA degradation can lead to lower UMI detection. In our study, the sequencing depth was standardized across all samples to minimize technical variability but differences in tissue preparation methods may still contribute to the observed variance in UMI counts.

### Inferred IHC for important neuropathological markers

**Fig. 2:**
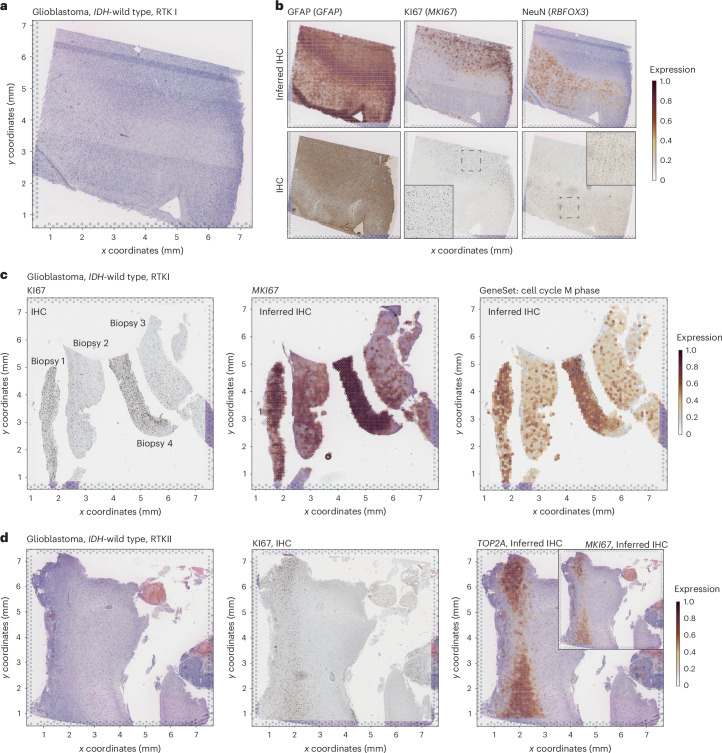
Inferred IHC. **a**, Representative H&E staining of GB RTKI sample (*n* = 1 participant). **b**, Top: comparison of inferred IHC from spatially resolved transcriptomics to super-resolution from GFAP, Ki67 and NeuN. The color intensity demonstrates the gene expression. Bottom: the aligned IHC stainings are presented from consecutive sections. **c**, Representative Ki67 staining of multiple biopsies from a participant with GB. Middle: the different proliferation indices across biopsies can be detected by the inferred IHC reconstruction. Right: quantification of mitosis-related gene expression allowed detailed exploration of the cell-cycle phases that aligns with the Ki67 staining (*n* = 1 participant). **d**, Representative H&E and Ki67 staining of GB RTKII sample. Left: on a gene expression level, the proliferation markers *TOP2A* and *MIK67* indicate a robust detection of highly proliferative regions (*n* = 1 participant). Paired IHC and spatial transcriptomics (for analysis of **a**–**d**) was available for 12 participants. Statistical testing was performed on all samples.

**Extended Data Fig. 1:**
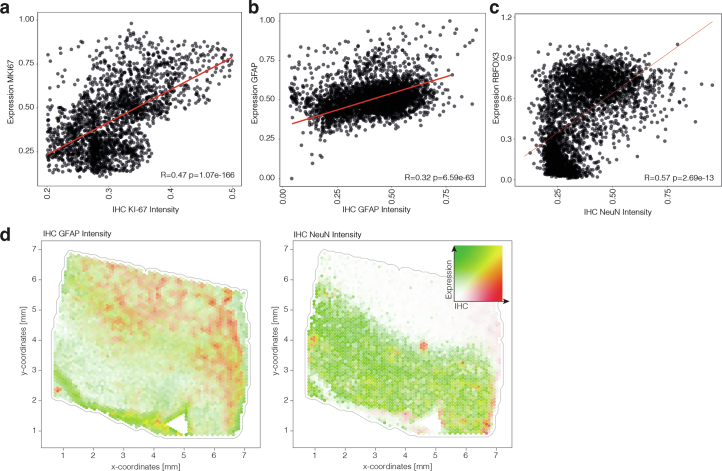
Analysis of inferred IHC. (**a**–**c**) Scatter plot of the smoothed image IHC intensity signal and the spatial expression abundance after co-registration and signal binning per spot. Statistical significance was assessed using a two-sided Pearson correlation test without adjustment for multiple comparisons **d**) Surface plots of the IHC and expression signal after co-registration and signal binning per spot.

While gene expression and protein abundance can vary at the cellular level, pinpointing protein abundance at a spatial resolution across whole slides is crucial for neuropathological diagnostics^2^. We posited that, on a whole slide scale, gene expression might offer insights into protein levels, facilitating precise discernment of the presence or absence of protein markers. To this end, we devised an innovative computational module named ‘inferred IHC’. This module harnesses super-resolution spatial transcriptomics through the Bayesian inference^11^ to forecast protein abundance, rendering it a viable diagnostic surrogate for traditional IHC (Fig. 2a,b). When juxtaposed with consecutive participant sections, our virtual stainings exhibited robust alignment with IHC-derived results (Fig. 2b). A direct assessment between signal intensity and mRNA abundance revealed notable correlations, evident in routinely applied diagnostic markers such as Ki67 (*R* = 0.47, *P* = 1.07 × 10^−166^), glial fibrillary acidic protein (GFAP; *R* = 0.32, *P* = 6.59 × 10^−63^) and neuronal nuclei (NeuN; *R* = 0.57, *P* = 2.69 × 10^−13^) (Extended Data Fig. 1a–c). Despite these correlations, a direct quantitative comparison between IHC and gene expression data from Visium technology presents challenges because of differences in resolution (Extended Data Fig. 1d). IHC stainings provide high-resolution images that pathologists use for visual interpretation, offering a spatially precise, cell-level view of protein expression. In contrast, gene expression data from Visium captures transcriptomic information at a different resolution, which can complicate direct interpretation. This disparity in resolution scales means that, while our inferred IHC module offers promising results, it should be seen as a complementary tool rather than a direct replacement for traditional IHC. Yet, inferring an infinite number of stainings on one single slide overcomes the changes of displayed surface that comes with actual IHC slides on serial sections. A pivotal diagnostic tool in neuropathology is the proliferation index, which measures cell-cycle activity in malignant samples and distinguishes between regions of high and low tumor proliferation (Fig. 2c). We compared standard Ki67 staining to regional expressions of proliferation markers such as *MKI67* and *TOP2A* (Fig. 2d). By quantifying expression across all cell-cycle stages, we provide neuropathologists with a nuanced perspective on proliferation activity, surpassing the limitations of single-protein marker stainings. The importance of proliferation assessment in grading tumors according to the WHO classification underscores the necessity for a contextualized evaluation of this marker. Using a spatial-transcriptomics-based proliferation index offers a detailed understanding of tissue architecture and tumor cell dynamics.

### Predictions and validation of CNAs

**Fig. 3:**
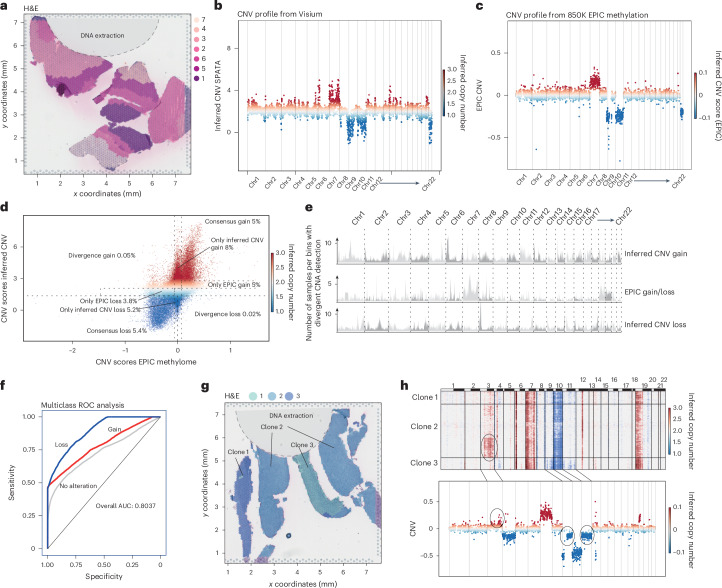
Inferred CNVs. **a**, H&E image of a GB sample (*n* = 1 participant). The gray area illustrates the punch position for the DNA extraction and 850K methylation analysis. **b**,**c**, Copy-number profile of the inferred CNV analysis (**b**) and the 850K methylation analysis (**c**). **d**, Scatter plot of the inferred CNV scores (*y* axis) and the 850K methylation scores (*x* axis). **e**, Mapping of the number of incorrect predictions of gains or losses across the chromosomes. The *y* axis indicates the number of participants with divergent predictions. The different gray scales mark even and uneven numbers of chromosomes. **f**, ROC analysis for predictions of gains and losses or diploid chromosome sets by inferred CNV scores. **g**, H&E image of a GB sample. The gray area illustrates the position from which the tissue was taken for the DNA extraction and 850K methylation analysis. **h**, CNV heat map illustrating the CNA heterogeneity across subclones. Bottom: the 850K methylation-based CNV profile. Analysis of CNV data was performed on the full cohort (107 participants).

**Extended Data Fig. 2:**
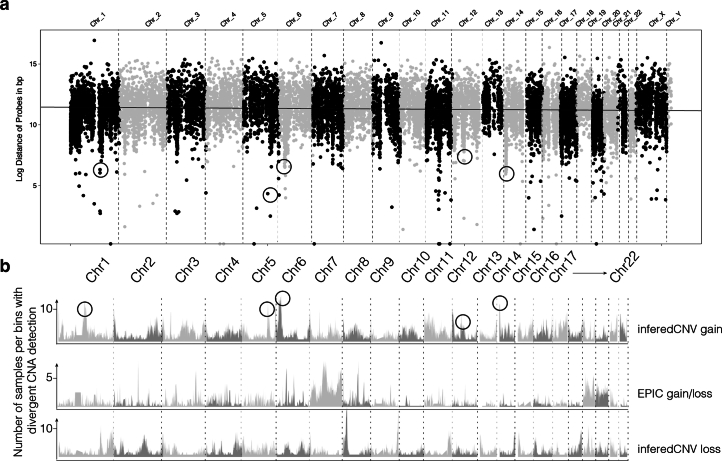
Inferred CNV: A detailed view. (**a**) Scatter plot of the probe-to-probe distance sorted by chromosomal positions along with the mismatch plot (Fig. 4) (**b**).

In addition to histopathological morphology and cellular staining, modern neuropathological diagnostics rely heavily on detailed genomic characteristics of CNS pathologies. Chromosomal alterations, such as the gain of chr7 and loss of chr10 in GB, along with the codeletion of chr1p and chr19q in oligodendrogliomas, are defining diagnostic criteria according to the WHO classification^2,12^. While inference of CNV from single-cell RNA sequencing is documented, recent advancements show that high-accuracy CNV profiles can be sourced from spatially resolved transcriptomics. Using an optimized algorithm for spatial transcriptomics, we computed CNV profiles for all participant samples. Validation against gold-standard 850K methylation assay (EPIC) CNV profiles revealed robust detection of CNAs, crucial for diagnosing the specific tumor type (Fig. 3a–c). Direct comparison of methylation-based CNV against our inferred CNV detection revealed a consensus of CNV alterations in 81.2% cases (consensus no alterations, 70.3%; consensus gains, 5.5%; consensus loss, 5.4%), only 0.05% divergent gains (gains detected by Visium and loss detected in the methylation-based CNV analysis) and 0.02% divergent losses (Fig. 3d). To more precisely examine the potential variability in accuracy of CNV detection across different chromosomal regions, we quantified and mapped the instances of mismatched detection (either gains or losses) from the inferred CNV calls across various participants along the chromosomes, demonstrating an increase in incorrectly detected gains in chr6p (Fig. 3e). To rule out that this effect is based on accumulation of specific cell types (for example, immune cells with higher expression of major-histocompatibility-complex-related genes on chr6p), we measured the abundance of spots with high chr6q copies (*n* = 10,000) against random spots without enriched chr6q (*n* = 10,000). For myeloid and lymphoid cells, we did not identify significant differences (tenfold cross-validation, *P* = 0.321).To further investigate the defined chromosomal regions with enhanced mismatches, we hypothesized that the probe design by 10X and the relative frequency of probes within chromosomal regions might lead to false predictions during gene binning. In other words, some chromosomal regions contain more probes than others. We measured the relative probe-to-probe distance and correlated this to the detected mismatches. Reduced probe-to-probe distance (in base pairs) was found to significantly correlate with the false detection of chromosomal alterations (*R* = 0.421, *P* = 2.43 × 10^−32^; Extended Data Fig. 2a,b).

Additionally, inferred CNV profiling leads to an underestimation of the gain of chr7, predominantly the centromeric region, while the start and telomeric regions of the chromosome are correctly predicted. We performed multiclass receiver operating characteristic (ROC) analysis to validate the accuracy of detecting gains and losses or diploid chromosome sets, demonstrating an overall area under the curve (AUC) of 80.37% (Fig. 3f). Notably, spatial resolution in the CNV profiles from spatial transcriptomics could discern the subclonal tissue architecture, a nuance missing in traditional methylation-based CNV profiles. For instance, DNA isolated from distinct tissue sections showed a CNV profile closely mirroring the profile inferred from spatial transcriptomics. However, some subclones with specific alterations were undetectable using methylation-based CNV methods and only identified with spatial transcriptomics (Fig. 3g,h). One limitation of inferred CNV profiles is their reduced chromosomal resolution, constraining precise predictions to larger chromosomal regions. Consequently, pinpointing single-gene amplifications or deletions, such as *EGFR*, *PDGFRA* or *CDKN2A/B*, is unattainable using only inferred CNV profiles. To overcome this limitation, we later investigate to what extent genotypes can be predicted by environmental phenotypes. Our findings underscore the capability of spatial transcriptomics to identify notable chromosomal alterations. Such revelations are pivotal in distinguishing between tumor types such as *IDH*-mutant astrocytoma and oligodendrogliomas, which are primarily discerned through their genomic makeup.

### Cell type distribution across tumor entities

**Fig. 4:**
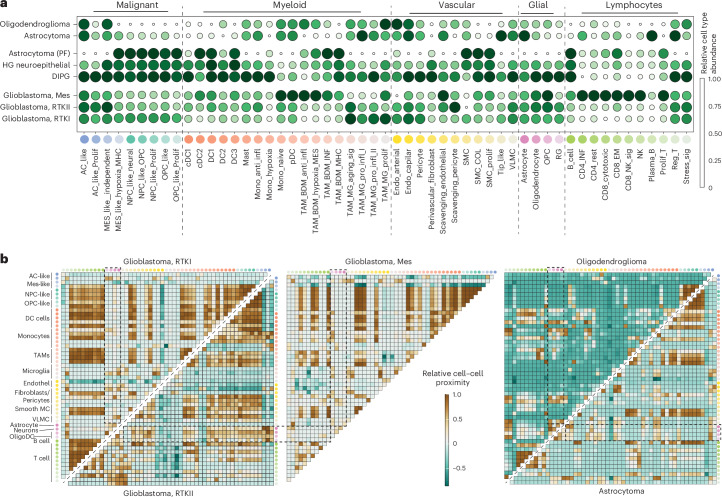
Cellular ecosystems of methylation classes. **a**, Overview of the relative cell type abundance across methylation classes. Cell type abundance was computed by the Cell2location algorithm and GBMap (annotation level 4) as a reference dataset. **b**, Left: heat map representation of the cell–cell interaction. Right: spatial proximity analysis across the different GB subclasses and *IDH*-mutant oligodendroglioma (top) and astrocytoma (bottom).

**Extended Data Fig. 3:**
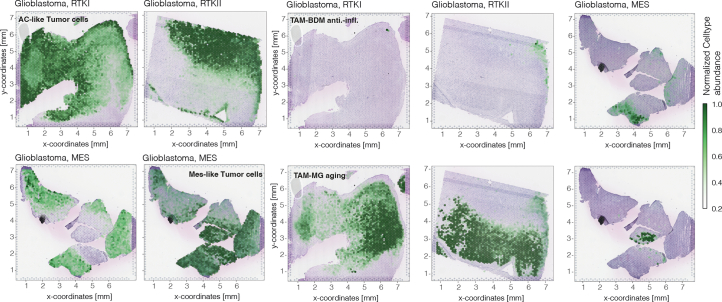
Representative enrichment scores. Surface plots of the enrichment of AC-like cells across GB subtypes. (upper pannel, left) Surface plot of the enrichment of tumor associated macrophages (type anti.-inflammatory) in the upper panel and aged associated microglia in the bottom part.

Traditional neuropathological diagnostics do not distinguish the distributions of immune, stromal and myeloid cells. However, recent research highlights that the prevalence of specific cellular subtypes, such as particular myeloid cells or cytotoxic T cells, can influence patient outcomes and therapeutic responses^13,14^. Given that future targeted therapies may rely on an in-depth understanding of cellular distributions, we investigated the potential of spatial transcriptomics to augment conventional neuropathological diagnostics by offering detailed cellular type and state distributions. Leveraging the robust and well-validated Cell2location algorithm, combined with an extensive GB reference dataset (GBMap), we predicted the abundance of myeloid, T cell and stromal subpopulations across all methylation classes (Fig. 4a). Although cell type distributions in GB were recently delineated^15^, less common subgroups, such as high-grade astrocytoma with piloid features or diffuse high-grade neuroepithelial tumors (adult-type, subgroup F)^16^, remain understudied. Although the tumor cell state distribution across GB subtypes was relatively equal with enrichment of the mesenchymal (Mes)-like states in the methylation group Mes, high-grade astrocytoma with piloid features lacked the astrocyte (AC)-like cell populations with significant enrichment of the Mes-like, oligodendrocyte-progenitor-like and neural-progenitor-like states (Fig. 4a). Comparing these rarer tumors to established GB methylation subgroups (receptor tyrosine kinase (RTK)I, RTKII and Mes), we found distinctive patterns. The methylation Mes subtype exhibited immune-rich microenvironments with an immunosuppressive myeloid profile, while others (RTKI and RTKII) displayed a notable absence of T cell and tumor-associated macrophage infiltration (Extended Data Fig. 3). Next, we computed the averaged cell proximity of all annotated cell types across GB subtypes demonstrating the different cell–cell interaction within individual subtypes (Fig. 4b). In line with the cell abundance, the epigenetic subgroups demonstrate distinct microenvironmental niches with dominating inflammatory response and neovascularization in the Mes subgroups and enhanced neuronal–tumor interaction in RTKII tumors. These findings are in line with different therapeutic response to anticonvulsive drugs^17^ or surgical strategies^18^. This diagnostic enhancement by including cellular deconvolution and microenvironmental niches offers unprecedented insights into the tumor architecture, an area often overlooked in traditional neuropathological assessments but of increasing relevance with emerging immune therapy approaches or treatment strategies in the cancer neuroscience field.

### Prediction of histological appearance and methylation subclasses

**Fig. 5:**
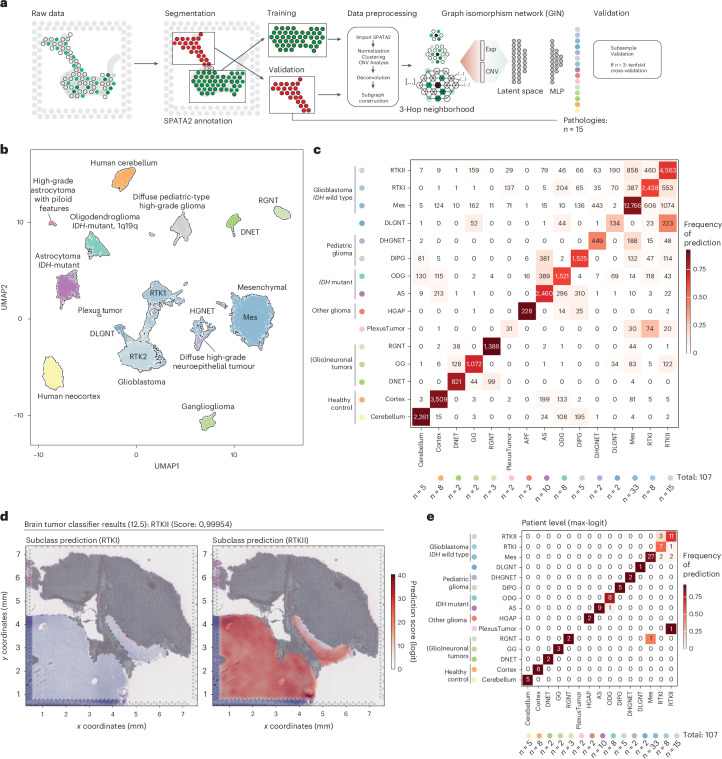
NePSTA performance on methylation subtype prediction. **a**, Schematic of the GNN workflow. **b**, Uniform manifold approximation and projection visualization of the GNN latent space embeddings, with colors denoting distinct epigenetic subclasses. **c**, Confusion matrix displaying accuracy in tumor subclass prediction; the ground truth is plotted against predictions on the subgraph level. **d**, For tumor-containing subgraphs, predictions of epigenetic subclasses are shown. A color map visualizes logits for RTKI and RTKII, revealing spatial nuances. **e**, Confusion matrix displaying accuracy in tumor subclass prediction; the ground truth is plotted against predictions on the subgraph level on a participant level.

In addition to histomorphological and genetic characterization, epigenetic classification has become an important tool for brain tumor classification, relying exclusively on molecular data. Recognizing the inability to glean epigenetic insights directly from spatial transcriptomics data, we posited that the spatial gene expression contours, influenced by both tumor and microenvironment, might sufficiently indicate methylation-based tumor subtypes using spatial transcriptomics data alone. To this end, we harnessed a GNN framework that leverages the breadth of local molecular data at each spot and its proximal neighborhood to predict histopathological phenotypes. Specifically, our model used three-hop subgraphs, where each node denoted a spatial location of the Visium array, interconnected by edges to neighboring and next neighboring locations (Fig. 5a).

To investigate the importance to integrate proximal signals we trained a *k*-hop network with *k* = {0,1,2,3}, where *k* = 0 represents a linear model. We trained the network to learn the spatial transcriptomic representation of the different tumor subclasses. The comparison (fivefold cross-validation) between the linear model and the GNNs (graph isomorphism network (GIN) or generative adversarial network (GAN)) showed a great increase in accuracy by integrating proximal signals. Furthermore, we demonstrated that for global max pooling, the GIN showed superior performance. The linear model without neighborhood information achieved an accuracy of 0.409, precision of 0.423, recall of 0.373 and *F*_1_ score of 0.388. In contrast, models incorporating neighborhood information, such as the GIN and GAN with one-hop, two-hop and three-hop neighborhoods, showed much higher performance. For instance, the GIN three-hop model achieved near-perfect metrics with an accuracy of 0.999, precision of 0.999, recall of 0.998 and *F*_1_ score of 0.998, while the GAN three-hop model achieved an accuracy of 0.995, precision of 0.991, recall of 0.995 and *F*_1_ score of 0.993. A GIN underpinned the GNN design, paired with a multilayer perceptron (MLP) for predictions, a structure previously shown to adeptly bridge local cellular graph analyses with broader patient phenotypes^19^. Input node features encompassed both gene expression and inferred CNV data, which were channeled through the MLP after embedding.

**Extended Data Fig. 4:**
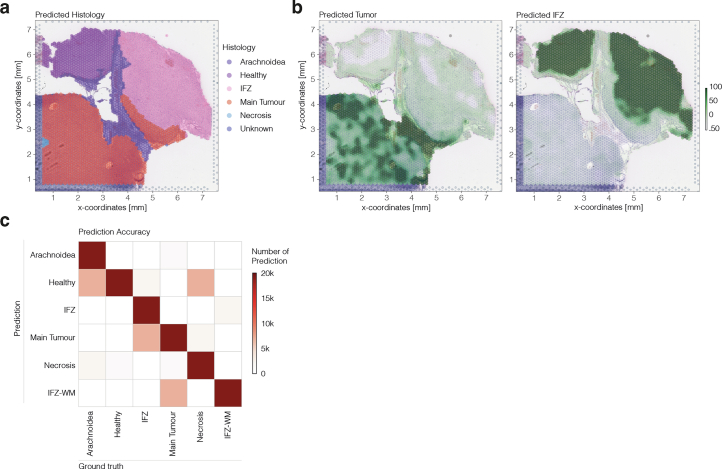
Regional prediction in graph-based neural networks. (**a**) Spatial transcriptomics overlay on a H&E image. Colors represent the supervised segmentation labels (n = 1 patient). (**b**) Predictive maps highlighting histological tumor areas (left) and infiltrative regions (right). The color spectrum indicates model-derived logits for each category. Predictive accuracy was tested on the full cohort (n = 107 patients). (**c**) Model’s predictive accuracy presented as a confusion matrix; ground truth on the x-axis versus predictions on the y-axis.

**Extended Data Fig. 5:**
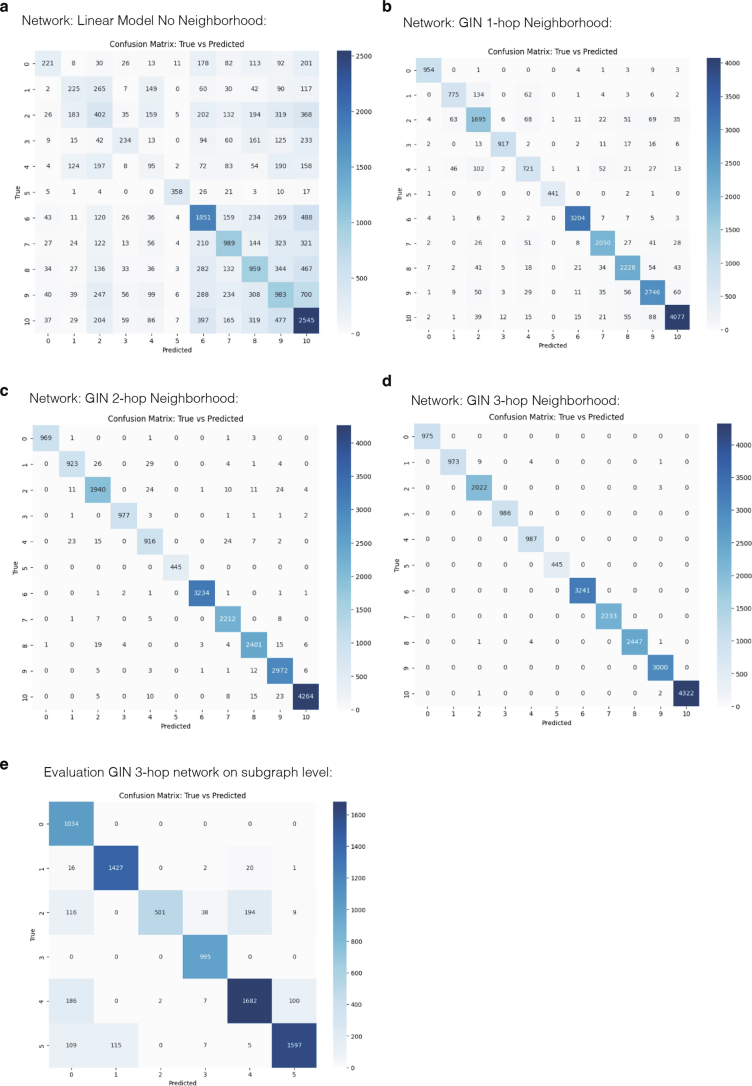
Graph-based neural networks of different neighborhood sizes. (**a**–**d**) Confusion matrix of the tumor subtype prediction of the full cohort (n = 107). **a**–**e** contain different sizes of neighborhoods (NN) starting from no-NN (a) to 1-hop(b) 2-hopn(c) and 3-hop (d). (**e**) Confusion matrix of the tumor subtype prediction of the full cohort (n = 107 patients) after 5-fold cross validation.

A pivotal step before subgroup prediction involved automated spatial transcriptomics data segmentation, aiming to classify subgraphs histologically. We used a tenfold cross-validation to train on *n* = 41 (cohort 1, samples with sufficient histological segmentation) participants to discern five core categories: main tumor, necrosis regions, infiltrative tumor regions, white matter and cortex. Nonmalignant sections were categorized as either healthy cortex or arachnoidea. To boost predictive accuracy, subgraphs from benign tissues (neocortex, hippocampus and cerebellum) were included. Model validation on *n* = 27 participants (samples with sufficient histological segmentation) from cohort 2 yielded an accuracy of 87.43% (ground truth: neuropathological segmentation) (Extended Data Fig. 4a–c). With the primary categorization established, we next analyzed whether the model could further subclassify regions classified as tumor into methylation subclasses. Specifically, we focused on subgraphs with at least one spot flagged as a tumor histologically and trained the model to predict methylation subtypes. We divided the samples into two distinct spatial transcriptomic datasets. For samples with multiple biopsies, we treated the raw data from each biopsy as separate datasets. In cases where the entire tissue was on a single slide, we segmented the samples as illustrated in Fig. 5a. To avoid bias from similar normalization and preprocessing in both the training and the validation cohorts, we processed each dataset individually after splitting the data at the count level. This strategy allowed us to validate our model on previously unseen data, despite the relatively low number of samples per histological subgroup. For tumor subgroups with more than three samples, we also conducted *n*-fold cross-validation (detailed data split information in Methods). Remarkably, the trained model demonstrated robust prediction on unseen data, with an accuracy of 0.893 at the participant level (precision, 0.873; recall, 0.877; *F*_1_ score, 0.870) (Fig. 5b–e). On GB and *IDH*-mutant glioma, we further performed participant-wise fivefold cross-validation to ensure robustness of the model demonstrating high performance (accuracy, 0.913; precision, 0.945; recall, 0.883; *F*_1_ score, 0.896) (Supplementary Table 1 and Extended Data Fig. 5a–d). To investigate sample heterogeneity, we showed that 60.8% of the cohort demonstrated the correct class in the majority of subgraphs (>80%), while 30.4% showed the correct subclass in at least 50% of the spots (Extended Data Fig. 5e). The overall accuracy of these analyses demonstrated not only that spatial transcriptomics data contain the same information for subtyping as bulk methylation data but that integrating proximal signals improves the classification accuracy of brain tumors (Extended Data Fig. 5a–d).

### Prediction of *MGMT* promoter methylation and *CDKN2A/B* loss

**Fig. 6:**
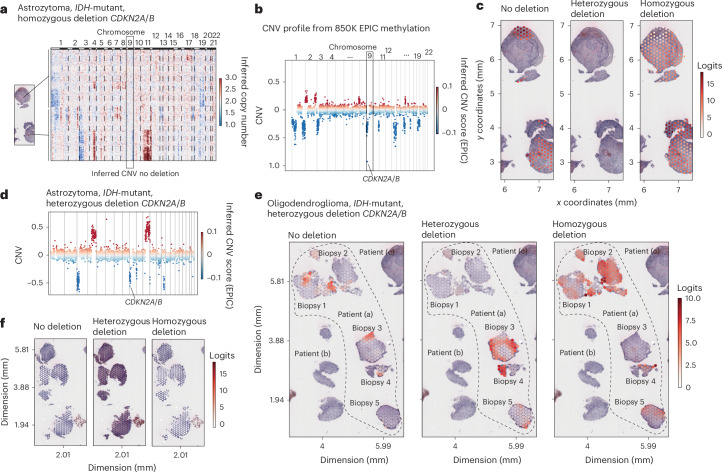
Graph-based deep learning for class prediction. **a**, Heat map of inferred CNVs of an *IDH*-mutant astrocytoma including the subclonal architecture with clonal gain in chr11 and partial loss of chr3. **b**, The loss *CDKN2A/B* was not detected by inferred CNV analysis. **c**, Spot-based prediction of *CDKN2A/B* loss revealing a detailed map of nondeleted regions (left) and loss of *CDKN2A/B* (right). **d**, Scatter plot of inferred CNVs of an *IDH*-mutant astrocytoma demonstrating the partial loss of chr9q and *CDKN2A/B*. **e**, Surface plots with prediction logits indicating the correct prediction of the heterozygous loss of *CDKN2A/B* across all biopsies of the participant. **f**, Surface maps of the prediction logits in a participant (including 5 biopsies) with heterozygous *CDKN2A/B* loss based on the EPIC methylation array. The prediction scores reveal a heterogeneous distribution of partial and full loss of *CDK2NA/B* within different biopsies and regions.

**Extended Data Fig. 6:**
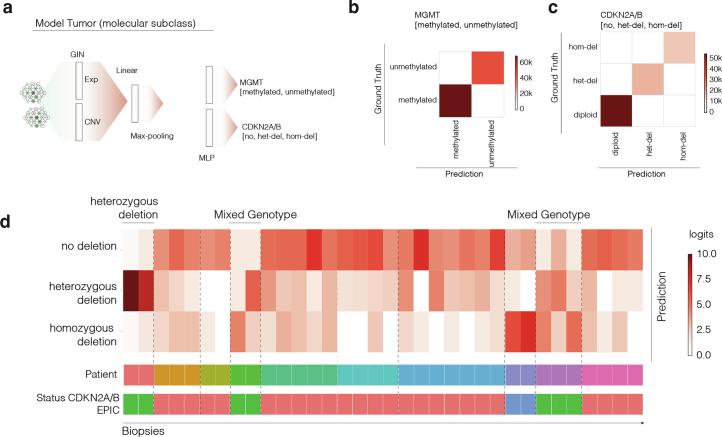
Heterogeneity of CDKN2A/B alterations. (**a**) Schematic of the GNN workflow Since the MGMT and CDKN2A/B status is available in both cohorts (n = 53 patients) we train (n = 30 patients) and evaluate (n = 23 patients) on separate cohorts. (**b**) Confusion matrix displaying accuracy in MGMT prediction; ground truth is plotted against predictions. (**c**) Confusion matrix of the CDKN2A/B prediction. (**d**) Heatmap of CDKN2A/B prediction scores across multiple low-grade glioma biopsies (n = 34 patients).

During our CNV analysis, we identified a limitation; the inferred CNV approach could not ascertain single-gene amplifications, notably for diagnostically relevant genes such as *EGFR* or *CDKN2A/B* (refs. ^2,12,20^). Moreover, discerning *MGMT* promoter methylation—a crucial clinical indicator^3^—proved challenging when relying solely on gene expression. Addressing this challenge, we postulated that the unique expression architecture inherent in tumors could potentially offer an opportunity to predict these molecular characteristics. To harness this insight, we augmented our GNN framework, incorporating multiple MLPs specifically designed to predict *MGMT* promoter methylation and the loss of *CDKN2A/B* (Extended Data Fig. 6a–c). By integrating spatial patterns of transcriptomic data, our enhanced model aimed to overcome the limitations of inferred CNV analysis, striving to achieve accurate predictions even when direct identification of molecular markers is not possible (single-gene deletions). For the *MGMT* promoter methylation, the deep-learning framework was able to predict the correct *MGMT* status in almost all cases, leading to an overall accuracy of 99% (*F*_1_ score, 1.0; precision and recall, 1.0). In inferred CNV, detection of *CDKN2A/B* loss and further differentiation between homozygous and heterozygous deletion were found to be impossible in cases when the loss was not associated with chr9p loss (Fig. 6a,b). Leveraging our GNN framework, we were able to differentiate between nondeleted control subgraphs and homozygous or heterozygous loss of *CDKN2A/B* with an accuracy of 85.4%. Furthermore, we performed whole-sample prediction to identify regional diversity of the *CDKN2A/B* deletion. Using each individual spot and associated subgraph, we computed the likelihood of *CDKN2A/B* loss, resulting in a spatial map of *CDKN2A/B* deletions (Fig. 6c). Regions without tumor abundance were correctly classified as nondeleted. Next, we evaluated an *IDH*-mutant astrocytoma with partial loss of *CDKN2A/B* (based on methylation-array data; Fig. 6d) demonstrating the highest prediction score for heterozygous deletion of *CDKN2A/B* across all four biopsies (Fig. 6e,f). As demonstrated in the two examples, tissue can be highly heterogeneous and regions of normal brain or myeloid infiltration can often affect the signal of chromosomal deletion, especially in morphologically lower-grade glioma in which the *CDKN2A/B* status is crucial for WHO grading and, even more with recent breakthroughs, currently restricted to specific grade^21^ for therapy decision.

### Microenvironmental differences between *CDKN2A-*mutant subclones

**Fig. 7:**
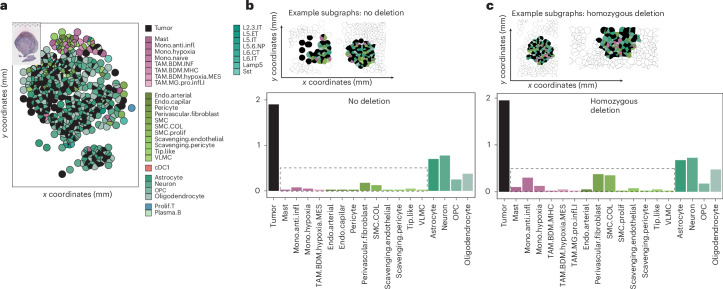
Ecosystems of *CDKN2A/B*-altered tumors. **a**, Example of the single-cell deconvolution of spatially resolved transcriptomic data using CytoSpace. **b**,**c**, Representative examples of subgraphs with high scores for no *CDKN2A/B* deletion (**b**) and subgraphs with high predictive scores for a homozygous *CDKN2A/B* deletion (**c**). Bottom: relative frequency plot of the abundance of cell type distribution between nondeleted and *CDKN2A/B-*deleted subgraphs.

To comprehensively assess the extent of *CDKN2A/B* loss and determine whether it is predominantly associated with homozygous or heterozygous deletion or whether it exhibits a heterogeneous distribution across tumor samples, we conducted an evaluation of all stereotactically obtained biopsies from *IDH*-mutant tumors (Extended Data Fig. 6d). Contrary to our expectations, our findings revealed that the majority of samples with heterozygous deletions (two of three) displayed a mixed genotype, encompassing both partial and complete loss of *CDKN2A/B* across different biopsy specimens. Given that the inferred CNV data lack specific details regarding *CDKN2A/B* alterations, we used the GNN architecture to elucidate the network’s ability to predict genotypic characteristics. Subsequent to performing single-cell deconvolution, we extracted and compared neighborhoods from subgraphs with the highest predictive scores for the absence of *CDKN2A/B* deletion and those indicative of complete *CDKN2A/B* loss. Although the relative abundance of tumor cells did not differ significantly between the two predicted genotypes, we observed substantial variations in microenvironmental composition (Fig. 7a–c). Specifically, neighborhoods with *CDKN2A/B* deletion exhibited an enriched presence of monocytes and endovascular cells, well in line with the typical co-occurrence of high-grade molecular (*CDKN2A/B* homozygous deletion) and morphological (angiogenesis and vascular proliferation, necrosis and perinecrotic reaction) findings.

## Discussion

The foundation of pathology lies in morphological evaluation but molecular markers have increasingly influenced prognostic stratification and treatment choices. The WHO’s CNS5 classification further emphasizes molecular markers as essential diagnostic criteria, particularly for tumor typing and subtyping. Methylation profiling, once used mainly for brain tumors, has become a powerful diagnostic tool across multiple tumor types. However, current methods of bulk DNA or RNA extraction can introduce sampling bias, missing subtle intratumoral variations. Despite the growing role of molecular markers, morphology remains indispensable, particularly for tumor grading, which often relies on morphological criteria. Molecular data alone can be misleading if sampled from heterogeneous tissue areas, where nontumor cells may dilute clinically important amplifications. In pathology, consistency across sections is critical but slight variations can occur, affecting diagnostic accuracy. New spatial technologies, such as spatial transcriptomics, offer an integrative solution by linking molecular data with morphology. Previously restricted to research, spatial sequencing now shows potential in routine diagnostics, as we demonstrated by validating a wide range of diagnostic markers in brain tumors. Incorporating spatial transcriptomics into neuropathological workflows enhances our understanding of tumor microenvironments, subclonal heterogeneity and cellular interactions. With advancing technology and reduced costs, spatial transcriptomics is likely to enable detailed analyses of larger, more complex tissue samples, offering cost effectiveness over traditional methods such as EPIC/panel-seq and IHC. Through machine learning, we mapped molecular predictions back onto histological images, assisting in the diagnostic process, particularly with limited tissue availability, which is common in brain tumors. Our framework captured spatial gene expression from FFPE and freshly frozen tissues, addressing RNA instability issues in FFPE samples. By applying GNNs, we improved diagnostic predictions through spatially resolved data. This approach aids in differentiating between tumor cells and reactive cells in high-proliferation areas, potentially avoiding grading inaccuracies. It also enables precise identification of key genetic deletions, such as *CDKN2A/B*, crucial for CNS5-compliant grading, which could otherwise be overlooked in bulk analyses.

Collectively, we established and validated NePSTA, a framework for comprehensive analysis of spatial transcriptomic analysis with a broad spectrum of diagnostic applications such as inferred IHC, CNAs and in-depth genotyping. We demonstrated that integration proximal signals of spatial transcriptomics along with AI-based algorithms can further predict epigenetic subgroups, perform automated segmentation and characterize prognostic genomic alterations such as *CDKN2A/B* loss at spatial resolution. We exemplified NePSTA with specifically challenging neuro-oncology samples that can readily be applied to increase diagnostic accuracy and precision. This alignment of morphology and molecular data unites the recently diverging fields of ‘traditional’ and ‘modern’ pathology, which both have their genuine and unique advantages, to jointly optimize patient care that ultimately depends on optimal diagnostic outcomes.

## Methods

### Clinical data and ethics

The tissue collection and processing were performed in accordance with the local ethic regulations (Institutional Review Board Heidelberg, S-318/2022) and the local ethics committee of the University of Freiburg approved the data evaluation, imaging procedures and experimental design (protocols 100020/09 and 472/15_160880). Written informed consent was obtained by all participants. Further information on research design is available in the Nature Portfolio Reporting Summary linked to this article.

### Spatial transcriptomics of FFPE samples

Preparation of FFPE samples for spatial transcriptomics followed protocols from 10X Genomics. Briefly, a small tissue section from a scored tissue block was rehydrated, cut into 5-µm sections and placed on a Visium slide’s capture frame. For stereotactic biopsies, multiple sections were included in a single frame. The sections were dried at 42 °C for 3 h and stored overnight in a desiccator. Paraffin removal involved sequential incubations in xylene, ethanol and water. The tissue was stained with hematoxylin and eosin (H&E), scanned with an Aperio AT2 slide scanner at ×40 and then decrosslinked in TE buffer (pH 9.0) at 70 °C for 1 h. The 10X Genomics V1 human transcriptome probe set was used for mRNA detection. After a 20-h hybridization at 50 °C, excess probes were removed through washes, ligated probes were retained and RNA digestion released the probes. Spatial barcodes were added through an extension reaction and an index PCR was performed on the basis of qPCR evaluation. Samples were sequenced on an Illumina NovaSeq6000, with targeted read depths of at least 100 million per sample.

### Spatial transcriptomics of freshly frozen samples

Freshly frozen samples were processed following 10X Genomics protocols. After cutting 5-µm sections, the tissue was placed in a Visium slide capture frame, incubated with methanol at −20 °C and then washed with isopropanol. H&E staining was applied, followed by imaging on an Aperio AT2 slide scanner. RNA release was achieved by permeabilization, spatial barcodes were added by reverse transcription and DNA was synthesized and amplified for library preparation. Sequencing was performed on a NextSeq500 with targeted read depths of at least 100 million reads per sample.

### Extraction of DNA for methylation analysis

For methylation analysis, DNA was extracted using the Maxwell RSC FFPE DNA purification kit. Tumor-rich regions were first identified by pathologists through examination of H&E-stained sections. A punch biopsy, either 1.5 mm or 3 mm in diameter, was then collected from the FFPE block in the selected area. The collected tissue was incubated with proteinase K at 70 °C overnight to remove paraffin and to digest the tissue. Following this, lysis buffer was added to release the DNA. The sample was subsequently cooled to allow the paraffin to solidify and the aqueous phase containing DNA was transferred to a new tube. DNA purification was then completed using the Promega Maxwell system and DNA concentrations were measured with the Qubit system (Invitrogen).

### Detection of DNA methylation

Methylation profiling and copy-number analysis were carried out using data from the Infinium MethylationEPIC BeadChip array (850K). Following the protocol established by Pfister et al.^22^, 250 ng of extracted DNA was subjected to bisulfite conversion with the Zymo EZ methylation kit. The DNA underwent repair using the Infinium HD FFPE restore kit, followed by amplification and hybridization onto an Infinium BeadChip. After several washing steps, single-base extension and fluorescent staining, the chip was scanned using an IScan software system. For bioinformatics, the minfi workflow (available from https://github.com/mwsill/minfi) was used for methylation analysis and conumee was used for copy-number calling (available from https://github.com/mwsill/conumee-2), following the methodology described by Sturm et al.^23^. Methylation classification was performed using version 12.5 of the classifier^24^.

### CNV detection

Most samples’ copy-number profiles were derived using the Infinium MethylationEPIC BeadChip array (850K), with four samples (GB5, GB14, GB15 and GB16) analyzed using the Infinium MethylationEPICv2.0 BeadChip array (935K). Sample processing was conducted at the neuropathology department in Heidelberg, following the procedure outlined in Pfister et al.^22^. Tumor-rich areas were identified on H&E-stained slides and DNA was extracted either from ten 10-µm-thick tissue slides or by punch biopsies from paraffin-embedded blocks (1.5 or 3 mm in diameter) using the Maxwell system (Promega). DNA quality was assessed using the Qubit system (Invitrogen) and 250 ng of DNA was then used for bisulfite conversion (Zymo). For methylation and CNV profiling, the samples were analyzed on either the Infinium MethylationEPIC or EPICv2.0 arrays (Illumina). Bioinformatic analyses, including methylation calling and CNV detection, were performed with minfi (available from https://github.com/mwsill/minfi) and conumee (available from https://github.com/mwsill/conumee-2), as described by Sturm et al.^23^, with classification performed using version 12.5 of the classifier^24^.

### IHC stainings

Immunohistochemical stainings were performed using a Ventana BenchMark Ultra Immunostainer. Stains for GFAP (mouse monoclonal, clone GA5; Cell Signaling), Ki67 (mouse monoclonal, clone MIB-1; Dako), ATRX (mouse monoclonal, clone BSB-108; Bio SB), Hip1R (rabbit, monoclonal, clone EPR9437; Abcam) and Vim (mouse monoclonal, clone V9; Dako) were conducted. ATRX, Hip1R and ATRX stainings were performed according to Sahm et al.^25^. GFAP and Ki67 were stained according to the protocol published by Wefers et al.^26^.

### Sample segmentation and histological annotation

Manual segmentation of the histological regions was performed by a neuropathologist according to the morphological features of the H&E scan. Spots were annotated using the loupe browser from 10X. A matrix containing the spatial barcode and the annotation of the neuropathologist was exported. The segmentation data frame was imported into the SPATA objects using the addFeature function of the SPATA2 package.

### Data import, preprocessing, filtering and normalization for spatial data analysis

For data analysis and quality control, we used the Cell Ranger pipeline from 10X Genomics. To facilitate spatial data analysis, we developed a custom framework. Data can be imported into our SPATA tool either by using a dedicated function (SPATA::initiateSpataObject_10X) or through manual entry of count matrices, barcode–coordinate matrices and H&E images. Standard import procedures include normalizing gene expression, which is achieved using the Seurat version 4.0 package. This process involves scaling transcript counts per spot to a total of 10,000, followed by natural log transformation. To control for batch effects, data normalization included regressing out sample batch variations and the percentage of ribosomal and mitochondrial gene expressions.

### CNA estimation

Our CNA analysis leverages a pipeline integrated within the SPATA2 R tool, with a development version available at https://github.com/theMILOlab/SPATA2. The SPATA2::runCnvAnalysis() command enables additional CNA analyses. Chromosomal bins were created using the SPATAwrapper::Create.ref.bins() function, with a bin size of 1 Mbp used for this study. Data were then rescaled and interpolated over a 10-kbp window, with normalization achieved using a loess regression model through SPATAwrappers::runCNV.Normalization().

### Multiclass ROC analysis

To evaluate the performance of our classification model in a multiclass setting, we used multiclass ROC analysis. This method extends the traditional binary ROC analysis to handle more than two classes by averaging pairwise comparisons between classes. The algorithm we used for this analysis was developed by Hand and Till^27^. This approach calculates the AUC by considering each class against all other classes and then averaging these pairwise AUC values. This method ensures that the simplicity of the AUC metric is maintained while providing a comprehensive evaluation for multiclass classification problems. The steps for the multiclass ROC analysis start with a pairwise comparison. For each class *k*, the AUC for the binary classification of class *k* is computed versus all other classes. Next, the AUC values obtained from all pairwise comparisons are averaged to get the overall multiclass AUC. We implemented this algorithm using the pROC library in R, which provides functions to compute multiclass ROC curves and AUC values^27^.

### Spatial autocorrelation Moran’s I

We assessed the spatial dependencies among spots using Moran’s I statistics. This analysis discerns whether gene expression patterns across the sample are spatially clustered, randomly distributed or dispersed. Moran’s I is indicative of spatial clustering when positive and spatial dispersion when negative. Moran’s I index is given by the following formula:

$$I=\frac{N}{W}\times \frac{{\sum }_{i}{\sum }_{j}{w}_{ij}({X}_{i}-\bar{X}\,)({X}_{j}-\bar{X}\,)}{{\sum }_{i}{({X}_{i}-\bar{X}\,)}^{2}},$$

where $N$ is the total number of spatial spots, ${X}_{i}$ and ${X}_{j}$ are the gene expression values at spots *i* and $j$, respectively. $\bar{X}$ is the mean gene expression value across all spots. ${w}_{{ij}}$ represents the spatial weight between spots *i* and $j$. $W$ is the sum of all spatial weights ${w}_{{ij}}$. The computation of Moran’s I, including the spatial weights matrix, was performed using the ‘inferSpatial.ac()‘ function from the SPATAwrappers package.

### Spatial correlation analysis

The hlpr_join_with_aes helper function was used to merge these data, with optional normalization for gene sets (method_gs) and smoothing (smooth), including adjustment of smoothing span (smooth_span) as needed. After data preparation, we constructed a graph representing spatial relationships among spots. This was achieved by generating a positional data frame (pos) containing the *x* and *y* coordinates linked to each spot’s barcode. We then created a spatial weight matrix (weight) using the getSpatialNeighbors function from the MERINGUE package, which computes proximity between spots. To identify primary spatial patterns across features, MERINGUE calculates a spatial cross-correlation index (SCI). This index assesses the correlation between gene pairs exhibiting significant spatial heterogeneity, considering genes expressed by a sufficient fraction of cells. The SCI is determined using the following formula:

$${\rm{SCI}}=\frac{N}{2\mathop{\sum }\nolimits_{i}^{N}\mathop{\sum }\nolimits_{j}^{N}{W}_{{ij}}}\frac{\mathop{\sum }\nolimits_{i}^{N}\mathop{\sum }\nolimits_{j}^{N}{W}_{{ij}}\left({x}_{i}-\bar{x}\right)\left({y}_{j}-\bar{y}\right)}{\sqrt{\mathop{\sum }\nolimits_{i}^{N}{\left({x}_{i}-\bar{x}\right)}^{2}}\sqrt{\mathop{\sum }\nolimits_{j}^{N}{\left({y}_{j}-\bar{y}\right)}^{2}}},$$

where *x*_*i*_ and *y*_*j*_ represent the levels of two different features and $\bar{x}$ and $\bar{y}$ are the mean feature levels. *W*_*ij*_ represents the spatial weight matrix or spatial weights between locations *i* and *j*. This matrix captures the spatial relationships or proximity between the spatial units (for example, barcodes or regions) in the spatial grid. The values in the spatial weight matrix *W*_*ij*_ quantify the proximity of spots. The feature matrix, feature_mat, contained the expression values of the features for each barcode and was transposed to align with the spatial weight matrix. The resulting matrix encapsulates the spatial correlation coefficients for each pair of features across the spatial grid, providing insights into the spatial distribution and coexpression patterns of the genes of interest.

### Imaging IHC and spatial transcriptomic quantification

To quantify the correlation between IHC staining from an image and the expression levels of the corresponding gene, we implemented a detailed image processing and analysis pipeline. We start by converting the IHC image to grayscale by extracting the blue-channel (img[,,3]) and transform into data.frame format using the reshape2::melt() function. The pixel intensity values were extracted and rescaled to a range of 0–1. Next, we performed a Gaussian kernel smoother:

$$\widehat{z}\left(x,y\right)=\frac{\mathop{\sum }\nolimits_{i=1}^{n}{z}_{i}\exp \left(-\frac{{\left(x-{x}_{i}\right)}^{2}+{\left(\,y-{y}_{i}\right)}^{2}}{2{\sigma }^{2}}\right)}{\mathop{\sum }\nolimits_{i=1}^{n}\exp \left(-\frac{{\left(x-{x}_{i}\right)}^{2}+{\left(\,y-{y}_{i}\right)}^{2}}{2{\sigma }^{2}}\right)},$$

where *z*_*i*_ represents the intensity value at the position (*x*_*i*_, *y*_*i*_) and $\hat{z}(x,\,y)$ is the smoothed intensity value at the spatial position (*x*, *y*). Next, we integrated the spatial distribution of the IHC intensity values (smoothed) and spatial transcriptomic gene expression of the corresponding genes. For the alignment of spatial data, we segmented the spatial data grid into smaller sections and calculates the mean expression value within each segment using the point.in.polygon method of the sf package. If ${y}_{i}\le y < {y}_{i+1{\rm{modn}}}$ or ${y}_{i+1{\rm{modn}}}\le y < {y}_{i}$, the *x* coordinate of the intersection of the ray extending to the right from (*x*, *y*) is computed with the following edge:

$${x}_{\rm{intersect}}={x}_{i}+\frac{((\,y-{y}_{i})\,({x}_{\{i+1{\rm{modn}}\}}-{x}_{i}))}{(\,{y}_{\{i+1{\rm{modn}}\}}-{y}_{i})}$$

where *x*_*i*_ and *y*_*i*_ represent the coordinates of the current vertex of the polygon. If no cells are present in a segment, a value of ‘NA’ is returned. To assess the correlation between image intensities and gene expression, we fitted a linear model to the data, with expression as the dependent variable and cell density as the independent variable. The correlation coefficient was calculated to quantify the relationship between image intensity and expression. The significance of the correlation was assessed using the *P* value from the correlation test.

### Single-cell deconvolution with Cell2location

To set up the Cell2location model, we configured the AnnData object using the setup_anndata function, defining parameters such as the number of cells per location and detection sensitivity. We trained the model on a graphics processing unit for 500 iterations to ensure computational efficiency. After training, we used the export_posterior function to extract the posterior distribution of cell type proportions, sampling 1,000 times for precise estimation across the spatial framework. Median estimates of cell type abundance were saved in adata_vis.obsm[‘q05_cell_abundance_w_sf’]. Finally, we incorporated the cell type abundance data back into the SPATA object using the addFeature function in SPATA2, allowing for further analysis steps within the spatial data framework.

### Spatial super-resolution inference with BayesSpace

Our analysis incorporated a super-resolution approach implemented into the runSuperresolution function, specifically designed to augment the resolution of spatial gene expression. We used the BayesSpace^11^ algorithm, which inferred super-resolution by performing enhanced clustering on principal component (PC) space derived from log-normalized gene expression data and then mapping the high-resolution PCs back to gene expression space using predictive models, such as linear and XGBoost regression, to spatially visualize and analyze refined gene expression patterns at a finer scale. To achieve this from SPATA2 data, we then created a SingleCellExperiment (SCE) object. The preprocessing of the spatial data was conducted through the BayesSpace::spatialPreprocess function, which is designed for Visium platform data. The data were then clustered spatially with the BayesSpace::spatialCluster function, where the number of clusters was set on the basis of our earlier determined unique features count. The clustering process was iterated for repetitions with a burn-in of iterations to ensure model stability and convergence. Following spatial clustering, we applied a super-resolution enhancement using BayesSpace::spatialEnhance, which further refines the spatial resolution of clusters by enhancing the signal within the spatial data.

### Inferred IHC visualization

For visualizing inferred IHC results, we used the plotInferredIHC (available from https://github.com/heilandd/NePSTA) function. This function is designed to create illustrative representations of the spatial distribution of cellular features within the tissue on the basis of the enhanced-resolution data. The visualization process began with the extraction of enhanced PC analysis dimensions from the sce.enhanced object and reference PC analysis dimensions from the SCE object using the reducedDim function. We then aligned the dimensions of the enhanced and reference datasets to a common number of PCs. With the BayesSpace::featurePlot function, we generated data for feature visualization on the basis of the enhanced PC analysis results. We normalized the feature intensities using the SPATA2::hlpr_normalize_vctr function to ensure that the intensity values were proportional and visually interpretable. Depending on the chosen type of visualization, either scatter plots or Voronoi tessellation were used to depict the spatial feature data. Scatter plots were rendered using the scattermore::geom_scattermore function, while Voronoi tessellation was executed with ggforce::geom_voronoi_tile. This allowed for a flexible representation of spatial data, either as individual data points or as contiguous spatial regions. The plots were further refined by adjusting the spatial coordinates to the range of the image and scaling the feature intensities for optimal visualization. The final output was a plot that combines the spatial coordinates with the feature data, overlaid onto the spatial image.

### Construction of spatial graphs from Visium spatially resolved transcriptomic data

Each spatial transcriptomic dataset was preprocessed using SPATA2, which included log-transforming the count matrix and aligning the imaging data (H&E Image). Nucleus positions were annotated through an automated ilastik pretrained segmentation algorithm. For samples where high imaging quality was lacking and automated segmentation was unsuccessful, we applied a modified version of a recently published CytoSpace algorithm, as described above. We extracted spot coordinates using the getCoordsDf function from the SPATA2 package and calculated a pairwise distance matrix representing spatial distances between all cell pairs on the basis of their *x* and *y* coordinates. To prevent computational issues from zero-distance values (self-distances), we substituted these values with a constant of 1,000, ensuring no cell was mistakenly marked as its own neighbor in later steps. We set a distance threshold slightly above the smallest nonzero value in the modified matrix to establish cell adjacency, where cells within this threshold were marked as adjacent (1) and those beyond as nonadjacent (0). The adjacency matrix was converted into an undirected graph using the graph_from_adjacency_matrix function from the igraph package, with rows and columns labeled by each cell’s unique barcode from getCoordsDf. We retrieved the gene expression matrix from the ‘obj’ object, transposing it to match the graph vertices. The matrix was then filtered to include only the top 5,000 variable genes, corresponding to labeled graph vertices, creating a combined structure of spatial and expression data. With this graph, we analyzed local spatial gene expression patterns by examining the neighborhood around specific query spots. A three-hop neighborhood for a query spot included all spots within three edges, capturing the spatial context and connectivity within the defined distance.

### The NePSTA graph neural network

The NePSTA framework uses GNNs to analyze spatially resolved multiomics data, integrating clinical, histological and molecular features. Using a GIN architecture, NePSTA models local spatial structures and predicts tumor-related parameters with high accuracy. A detailed description of the network is provided on GitHub (https://github.com/heilandd/NePSTA).

#### Data splitting and preparation

From 107 EPIC-characterized participants, datasets were divided into training and validation subsets. For multibiopsy samples, datasets were split by biopsy cores; for single-specimen samples, manual segmentation was performed using SPATA2 to create regions. Training data comprised 97,000 subgraphs from tumor datasets and 12,000 subgraphs from healthy controls, created using a three-hop neighborhood approach. Evaluation data comprised subgraphs from the validation datasets covering a range of epigenetic classes for robust model evaluation. A detailed description of the network is provided on GitHub (https://github.com/heilandd/NePSTA).

### Node and edge features

Expression profiles (top 5,000 genes), CNAs, histological annotations (one-hot encoded) and encoded H&E image vectors were included in the neural networks. Edge features were defined by spatial proximity with up to six neighbors per node; self-loops ensured that nodes retained the original feature information. A detailed description of the network is provided on GitHub (https://github.com/heilandd/NePSTA).

### NePSTA GNN architecture

The back bone consisted of a three-layer GIN to process subgraphs, aggregating node features using MLPs and message-passing techniques. Regularization consisted of batch normalization, dropout layers and leaky rectified linear unit activations to stabilize training. The latent space consisted of a global mean pooling of features to enable graph-level predictions for survival and tumor classification. A detailed description of the network is provided on GitHub (https://github.com/heilandd/NePSTA).

### Evaluation metrics

#### Metrics used

Accuracy, precision, recall, *F*_1_ score and confusion matrices were used to evaluate performance. Loss functions consisted of cross-entropy loss for categorical variables and *L*_1_ norm and mean squared error for continuous variables. Losses were combined using weighted sums that were optimized during training. A detailed description of the network is provided on GitHub (https://github.com/heilandd/NePSTA).

### Training and inference

#### Optimization

The Adam optimizer was used to minimize task-specific losses over epochs. Gradients were reset after each batch to refine model weights iteratively. A detailed description of the network is provided on GitHub (https://github.com/heilandd/NePSTA).

### Evaluation of the subgraph cell composition

To reconstruct the cellular composition of each subgraph, we pinpointed cellular positions as previously described and then determined the probable cellular composition by considering the cell count per spot and the deconvolution scores from Cell2location, using the spAnchor package for implementation. We began by pinpointing each nucleus’s spatial location using the SPATAwrappers getNucleusPosition function and recorded the spot coordinates using the SPATA2 getCoordsDf function. The spatial coordinates representing the positions of nuclei were obtained as $P=\{\,{{{p}}}_{{{i}}}|i=1,\,\ldots ,\,N\,\}$, where *p*_*i*_ is the coordinate pair for the *i*th nucleus and *N* is the total number of nuclei. Spatial grid coordinates corresponding to the transcriptomics data points were retrieved, denoted as $G=\{{{{g}}}_{{{j}}}| \,j=1,\ldots ,M\,\}$, with each *g*_*j*_ representing the coordinate pair for the *j*th grid point. For each grid point *g*_*j*_, a vector of deconvolution scores ${{{D}}}_{{{j}}}=\{{{{d}}}_{{{jk}}}| k=1,\ldots ,T\,\}$ was extracted, where *d*_*jk*_ represents the score for the *k*th cell type at grid point *j* and *T* is the number of cell types. The scores were normalized to a range of [0, 1] and the number of cells of each type at each grid point was estimated as follows:

$${{{C}}}_{{{jk}}}={\rm{round}}\left(\frac{{{{d}}{\prime} }_{{{jk}}}\times {{{N}}}_{{{j}}}}{{\sum }_{{{k}}=1}^{{{T}}}{{{d}}{\prime} }_{{{jk}}}}\right)$$

where ${{{d}}{\prime} }_{{{jk}}}$ is the normalized score and *N*_*j*_ is the number of cells at grid point *j*. Cell types were assigned to each grid point *g*_*j*_ to create a mapping *M*_*j*_, correlating grid points with their respective cell types. The cell type mapping was integrated with nucleus position data to produce a comprehensive spatial map of cell type distribution: $S=\{({{{p}}}_{{{I}}},{{{M}}}_{{{j}}}){|{{p}}}_{{{i}}}\in P,{{{M}}}_{{{j}}}\in M\,\}$. This methodology facilitates the visualization and analysis of the cellular composition within the tissue section, providing insights into the complex spatial organization of the cellular environment.

### Statistics and reproducibility

No statistical method was used to predetermine sample size. No data were excluded from the analyses. The experiments were not randomized. The investigators were not blinded to allocation during experiments and outcome assessment. Data distribution was measured and tailored methods were applied to handle non-normal distributions in gene expression, methylation and image data.

### Reporting summary

Further information on research design is available in the Nature Portfolio Reporting Summary linked to this article.

## Supplementary information

Reporting Summary

Supplementary Table Prediction metrices of the GAN.

## Source data

Source Data Raw data of the figures and participant characteristics.

## Data Availability

The spatial transcriptomics data (validation cohort) used in this study were deposited to Dryad (10.5061/dryad.h70rxwdmj)^28^ and Zenodo (10.5281/zenodo.14064047)^29^ and are accessible to the public. Source data are provided with this paper.

## Code availability

The version of our NePSTA pipeline used in this study can be accessed from GitHub (https://github.com/heilandd/NePSTA). SPATA2 can also be assessed from GitHub (https://themilolab.github.io/SPATA2/).
