# Supplementary material for: Spatially resolved transcriptomics and graph-based deep learning improve accuracy of routine CNS tumor diagnostics
Source: Nat Cancer. 2025 Jan 29;6(2):292–306. doi: 10.1038/s43018-024-00904-z (PMC11864981; doi:10.1038/s43018-024-00904-z)
Supplement: Supplementary file 1 — Reporting Summary [file 43018_2024_904_MOESM1_ESM.pdf]

nature portfolio

Corresponding author(s): Henrik Heiland

Last updated by author(s): Dec 4, 2024

Reporting Summary

Nature Portfolio wishes to improve the reproducibility of the work that we publish. This form provides structure for consistency and transparency in reporting. For further information on Nature Portfolio policies, see our Editorial Policies and the Editorial Policy Checklist.

Statistics

For all statistical analyses, confirm that the following items are present in the figure legend, table legend, main text, or Methods section.

n/a	Confirmed	
☐	☒	The exact sample size (*n*) for each experimental group/condition, given as a discrete number and unit of measurement
☐	☒	A statement on whether measurements were taken from distinct samples or whether the same sample was measured repeatedly
☐	☒	The statistical test(s) used AND whether they are one- or two-sided<br>*Only common tests should be described solely by name; describe more complex techniques in the Methods section.*
☐	☒	A description of all covariates tested
☐	☒	A description of any assumptions or corrections, such as tests of normality and adjustment for multiple comparisons
☐	☒	A full description of the statistical parameters including central tendency (e.g. means) or other basic estimates (e.g. regression coefficient) AND variation (e.g. standard deviation) or associated estimates of uncertainty (e.g. confidence intervals)
☐	☒	For null hypothesis testing, the test statistic (e.g. *F*, *t*, *r*) with confidence intervals, effect sizes, degrees of freedom and *P* value noted<br>*Give P values as exact values whenever suitable.*
☒	☐	For Bayesian analysis, information on the choice of priors and Markov chain Monte Carlo settings
☐	☒	For hierarchical and complex designs, identification of the appropriate level for tests and full reporting of outcomes
☐	☒	Estimates of effect sizes (e.g. Cohen's *d*, Pearson's *r*), indicating how they were calculated

Our web collection on statistics for biologists contains articles on many of the points above.

Software and code

Policy information about availability of computer code

Data collection

Data were collected using the Space ranger pipeline v3.1. Space ranger output were processed by SPATA2 v2.4

Data analysis

The version of our NePSTA pipeline used in this study can be accessed via https://github.com/heilandd/NePSTA. The used SPATA2 can be assessed here: https://themilolab.github.io/SPATA2/ The used packages and versions are listed here: attached base packages:

```
 other attached packages:
 [1] kableExtra_1.3.4   igraph_2.0.3   lubridate_1.9.3   forcats_1.0.0   stringr_1.5.1   dplyr_1.1.4
 [7] purrr_1.0.2   readr_2.1.5   tidyr_1.3.1   tibble_3.2.1   ggplot2_3.5.1   tidyverse_2.0.0
[13] reticulate_1.37.0   SPATA2_2.0.4   DeepSPATA_0.0.0.9000 devtools_2.4.5   usethis_2.1.6   shinyBS_0.61.1
[19] Biobase_2.60.0   BiocGenerics_0.46.0
loaded via a namespace (and not attached):
 [1] pheatmap_1.0.12   DBI_1.2.3   bslib_0.8.0   httr_1.4.7   registry_0.5-1
 [6] BiocParallel_1.33.11   prettyunits_1.2.0   minfi_1.46.0   bumphunter_1.42.0   yulab.utils_0.1.5
[11] ggplotify_0.1.2   RCDT_1.2.1   GenomicAlignments_1.36.0   sparseMatrixStats_1.11.1   brio_1.1.3
[16] spatstat.geom_3.3-2   pillar_1.9.0   phateR_1.0.7   R6_2.5.1   boot_1.3-30
[21] gsw_1.1-1   mime_0.12   scCustomize_1.1.1   uwot_0.1.16   genefilter_1.82.1
[26] Rttf2pt1_1.3.12   viridis_0.6.5   Rhdf5lib_1.21.0   SeuratData_0.2.2   ROCR_1.0-11
[31] Hmisc_5.0-1   rprojroot_2.0.4   ggpubr_0.6.0   downloader_0.4   limma_3.56.2
[36] nor1mix_1.3-0   parallelly_1.38.0   GlobalOptions_0.1.2   FNN_1.1.4   caTools_1.18.2
[41] polyclip_1.10-7   NMF_0.26   htmltools_0.5.8.1   NFCN2_0.0.0.9000   fansi_1.0.6
[46] e1071_1.7-14   googledrive_2.1.0   commonmark_1.9.1   Azimuth_0.4.6   remotes_2.4.2
[51] swfscMisc_1.6   ggrepel_0.9.5   classInt_0.4-10   car_3.1-2   spData_2.3.0
```

```
 [56] ComplexHeatmap_2.15.4    fgsea_1.26.0             snakecase_0.11.0         spatstat.utils_3.0-5     HDO.db_0.99.1
 [61] clusterProfiler_4.8.1    rpart_4.1.23             beanplot_1.3.1           clue_0.3-64              scatterpie_0.2.0
 [66] fitdistrplus_1.2-1       goftest_1.2-3            tidyselect_1.2.1         RSQLite_2.3.1            cowplot_1.1.3
 [71] GenomeInfoDbData_1.2.10  utf8_1.2.4               scattermore_1.2          sessioninfo_1.2.2        rvest_1.0.3
 [76] spatstat.data_3.1-2      gridExtra_2.3            fs_1.6.4                 sctransform_0.4.1        ohenery_0.1.1
 [81] RColorBrewer_1.1-3       future.apply_1.11.2      R.oo_1.25.0              RcppHNSW_0.6.0           rtracklayer_1.60.0
 [86] vipor_0.4.5              doRNG_1.8.6              furrr_0.3.1              Rtsne_0.17               DelayedMatrixStats_1.22.6
 [91] lazyeval_0.2.2           sass_0.4.9               scales_1.3.0             carData_3.0-5            munsell_0.5.1
 [96] treeio_1.24.1            R.utils_2.12.2           profvis_0.3.8            V8_4.4.2                 bitops_1.0-8
[101] R.methodsS3_1.8.2        KEGGREST_1.40.0          promises_1.3.0           shape_1.4.6              rhdf5filters_1.11.2
[106] terra_1.7-46             zoo_1.8-12               illuminaio_0.42.0        GenomicFeatures_1.52.0   randomcoloR_1.1.0.1
[111] locfit_1.5-9.7           DelayedArray_0.26.7      spdep_1.2-8              metR_0.14.0              Rvcg_0.22.1
[116] RSpectra_0.16-2          SeuratDisk_0.0.0.9020    assertthat_0.2.1         paletteer_1.6.0          tools_4.3.3
[121] ape_5.7-1                processx_3.8.1           shiny_1.9.0              BiocFileCache_2.7.2      SingleCellExperiment_1.22.0
[126] oce_1.8-0                fftwtools_0.9-11         rlang_1.1.4              generics_0.1.3           ggridges_0.5.6
[131] extrafont_0.19           corrplot_0.92            evaluate_0.24.0          GenomeInfoDb_1.36.0      fastcluster_1.2.3
[136] siggenes_1.74.0          BiocIO_1.10.0            stringdist_0.9.10        reshape2_1.4.4           colorspace_2.1-1
[141] ellipsis_0.3.2           data.table_1.15.4        withr_3.0.1              gargle_1.4.0             RCurl_1.98-1.12
[146] presto_1.0.0             confuns_1.0.3            restfulr_0.0.15          xtable_1.8-4             plyr_1.8.9
[151] lme4_1.1-35.5            aplot_0.2.3              systemfonts_1.1.0        mclust_6.0.0             httpuv_1.6.15
[156] rmarkdown_2.27           units_0.8-5              MASS_7.3-60.0.1          broom_1.0.6              deldir_2.0-4
[161] GO.db_3.17.0             sandwich_3.0-2           rhdf5_2.43.0             tensor_1.5               googlesheets4_1.1.0
[166] vctrs_0.6.5              lifecycle_1.0.4          readxl_1.4.2             proxy_0.4-27             codetools_0.2-19
[171] fastDummies_1.7.3        DT_0.33                  SPATAData_0.0.0.9000     SPATAImmune_0.0.0.9000   DDRTree_0.1.5
[176] nlme_3.1-164             future_1.34.0            progress_1.2.3           dbplyr_2.3.2             pkgload_1.3.2
[181] cellranger_1.1.0         jquerylib_0.1.4          Rcpp_1.0.13              shinydashboard_0.7.2     rstudioapi_0.16.0
[186] patchwork_1.2.0          stringi_1.8.4            miscTools_0.6-28         hms_1.1.3                pbapply_1.7-2
[191] minqa_1.2.7              multtest_2.56.0          cachem_1.1.0             tester_0.1.7             BiocManager_1.30.20
[196] hdf5r_1.3.11             tidytree_0.4.2           listenv_0.9.1            XVector_0.40.0           ggrastr_1.0.2
[201] urlchecker_1.0.1         plotly_4.10.4            WGCNA_1.72-1             ggtree_3.8.0             jpeg_0.1-10
[206] enrichplot_1.20.0        GetoptLong_1.0.5         pkgbuild_1.4.0           ggfun_0.1.5              HDF5Array_1.28.1
[211] htmlwidgets_1.6.4        Formula_1.2-5            class_7.3-22             memoise_2.0.1            crayon_1.5.3
[216] gridGraphics_0.5-1       Seurat_5.0.3             rappdirs_0.3.3           S4Arrays_1.2.1           xml2_1.3.4
[221] filelock_1.0.2           preprocessCore_1.62.1    GOSemSim_2.26.0          s2_1.1.7                 png_0.1-8
[226] progressr_0.14.0         tzdb_0.4.0               MALDIquant_1.22.1        fastmap_1.2.0            coda_0.19-4
[231] tidygraph_1.2.3          pkgconfig_2.0.3          cli_3.6.3                beeswarm_0.4.0           DOSE_3.26.1
[236] ggforce_0.4.2            ps_1.7.5                 ggsignif_0.6.4           nnet_7.3-19              shinyWidgets_0.8.6
[241] gridBase_0.4-7           SummarizedExperiment_1.30.2 ggalluvial_0.12.5     maxLik_1.5-2             lmtest_0.9-40
[246] RcppAnnoy_0.0.22         timechange_0.3.0         viridisLite_0.4.2        askpass_1.2.0            foreign_0.8-86
[251] splines_4.3.3            blob_1.2.4               annotate_1.78.0          reshape_0.8.9            impute_1.74.1
[256] XML_3.99-0.15            network_1.18.1           globals_0.16.3           ggbeeswarm_0.7.2         knitr_1.48
[261] ggprism_1.0.4            ica_1.0-3                spam_2.10-0              stats4_4.3.3             compiler_4.3.3
[266] LaplacesDemon_16.1.6     rjson_0.2.21             sf_1.0-16                shinybusy_0.3.3          janitor_2.2.0
[271] anndata_0.7.5.6          biomaRt_2.56.0           extrafontdb_1.0          grid_4.3.3               bit_4.0.5
[276] BiocNeighbors_1.17.1     lsa_0.73.3               SPATAwrappers_0.0.0.9000 glue_1.7.0               sp_2.1-4
[281] ggnetwork_0.5.12         digest_0.6.36            quadprog_1.5-8           irlba_2.3.5.1            leiden_0.4.3.1
[286] tiff_0.1-11              base64_2.0.1             graphlayouts_1.0.0       rgl_1.1.3                magick_2.8.4
[291] GenomicRanges_1.52.0     foreach_1.5.2            spatstat.random_3.3-1    zlibbioc_1.46.0          dotCall64_1.1-1
[296] tweenr_2.0.3             CellChat_1.6.1           lattice_0.22-5           openssl_2.2.0            ggraph_2.1.0
[301] rsvd_1.0.5               gson_0.1.0               nloptr_2.1.1             yaml_2.3.10              qvalue_2.32.0
[306] later_1.3.2              statnet.common_4.9.0     backports_1.5.0          rstatix_0.7.2            shadowtext_0.1.2
[311] Rsamtools_2.15.2         AnnotationDbi_1.62.1     parallel_4.3.3           rematch2_2.1.2           sna_2.7-1
[316] miniUI_0.1.1.1           gtable_0.3.5             abind_1.4-5              xfun_0.46                Cairo_1.6-0
[321] webshot_0.5.5            Biostrings_2.68.0        curl_5.2.1               callr_3.7.3              GEOquery_2.68.0
[326] doParallel_1.0.17        dynamicTreeCut_1.63-1    KernSmooth_2.23-22       survival_3.5-8           desc_1.4.2
[331] jsonlite_1.8.8           harmony_1.2.0            magrittr_2.0.3           scrime_1.3.5             svglite_2.1.2
[336] base64enc_0.1-3          monocle3_1.3.4           iterators_1.0.14         spatstat.univar_3.0-0    matrixStats_1.3.0
[341] Matrix_1.6-5             SeuratObject_5.0.2       testthat_3.1.8           EBImage_4.41.1           fastmatch_1.1-3
[346] checkmate_2.2.0          MatrixGenerics_1.12.3    gtools_3.9.5             shinyjs_2.1.0            SnowballC_0.7.1
[351] htmlTable_2.4.1          spatstat.sparse_3.1-0    rngtools_1.5.2           roxygen2_7.2.3           RANN_2.6.1
[356] SeuratWrappers_0.3.1     S4Vectors_0.38.1         wk_0.9.2                 circlize_0.4.15          hdWGCNA_0.3.03
[361] spatstat.explore_3.3-1   IRanges_2.34.0           bit64_4.0.5              cluster_2.1.6            farver_2.1.2
[366] zip_2.3.0                gplots_3.1.3.1
```

For manuscripts utilizing custom algorithms or software that are central to the research but not yet described in published literature, software must be made available to editors and reviewers. We strongly encourage code deposition in a community repository (e.g. GitHub). See the Nature Portfolio guidelines for submitting code & software for further information.

Data

Policy information about availability of data

All manuscripts must include a data availability statement. This statement should provide the following information, where applicable:

- Accession codes, unique identifiers, or web links for publicly available datasets
- A description of any restrictions on data availability
- For clinical datasets or third party data, please ensure that the statement adheres to our policy

The spatial transcriptomics data (validation cohort) used in this study has been deposited on DataDryad and is accessible to the public (DOI: 10.5061/dryad.h70rxwdmj) and Zenodo: https://doi.org/10.5281/zenodo.14064047

Research involving human participants, their data, or biological material

Policy information about studies with human participants or human data. See also policy information about sex, gender (identity/presentation), and sexual orientation and race, ethnicity and racism.

Reporting on sex and gender	We did not include analysis on sex-specific questions
Reporting on race, ethnicity, or other socially relevant groupings	We did not include analysis addressing differences in race or ethnicity
Population characteristics	Patients characteristics are provided in the source data including diagnosis, Age and Sex.
Recruitment	We recruited patients who were treated in the included centres. No defined recruitment strategy was included.
Ethics oversight	University of Freiburg and Heidelberg, Ethic committee, Written informed consent was obtained by all participants

Note that full information on the approval of the study protocol must also be provided in the manuscript.

Field-specific reporting

Please select the one below that is the best fit for your research. If you are not sure, read the appropriate sections before making your selection.

☒ Life sciences ☐ Behavioural & social sciences ☐ Ecological, evolutionary & environmental sciences

For a reference copy of the document with all sections, see nature.com/documents/nr-reporting-summary-flat.pdf

Life sciences study design

All studies must disclose on these points even when the disclosure is negative.

Sample size	In this study we report a total of 130 samples
Data exclusions	No data were exluded
Replication	No biological or technical replicates were used
Randomization	We did not include randomization
Blinding	We did not include blinding.

Reporting for specific materials, systems and methods

We require information from authors about some types of materials, experimental systems and methods used in many studies. Here, indicate whether each material, system or method listed is relevant to your study. If you are not sure if a list item applies to your research, read the appropriate section before selecting a response.

Materials & experimental systems

n/a	Involved in the study
☐	☒ Antibodies
☒	☐ Eukaryotic cell lines
☒	☐ Palaeontology and archaeology
☒	☐ Animals and other organisms
☒	☐ Clinical data
☒	☐ Dual use research of concern
☒	☐ Plants

Methods

n/a	Involved in the study
☒	☐ ChIP-seq
☒	☐ Flow cytometry
☒	☐ MRI-based neuroimaging

Antibodies

Antibodies used

Antigen GFAP Manufacturer:Ventana Roche Cat-Number:760-4256 Dilution1:2.000
Antigen Hip1R Manufacturer:Abcam Cat-Number:140608 Dilution 1:200
Antigen Ki67 Manufacturer:Dako Cat-Number:M7240 Dilution 1:100
Antigen ATRX Manufacturer:Bio SCat-Number:B BSB3296 Dilution1:2.000
Antigen NeuN Manufacturer:Merck Millipore Cat-Number:MAB377 Dilution 1:250
Antigen Vim Manufacturer:Dako Cat-Number:M0725 Dilution 1:900

Validation

This antibodies are validated in a broad range of state-of-the-art neuropathology diagnostic. All protocols were established, validated and are continuously monitored in an accredited laboratory according to DIN EN ISO 17020

Plants

Seed stocks

NA

Novel plant genotypes

NA

Authentication

NA
