# Supplementary material for: Spatially resolved transcriptomics and graph-based deep learning improve accuracy of routine CNS tumor diagnostics
Source: Nat Cancer. 2025 Jan 29;6(2):292–306. doi: 10.1038/s43018-024-00904-z (PMC11864981; doi:10.1038/s43018-024-00904-z)
Supplement: Supplementary file 2 — Prediction metrices of the GAN. [file 43018_2024_904_MOESM2_ESM.pdf]

Model	Accuracy	Precision	Recall	F1 Score
Linear Model (no Neighborhood)	0.409	0.423	0.373	0.388
GIN 1-hop Neighborhood	0.914	0.909	0.9023	0.905
GIN 2-hop Neighborhood	0.981	0.978	0.977	0.978
GIN 3-hop Neighborhood	0.999	0.999	0.998	0.998
GAN 1-hop Neighborhood	0.874	0.865	0.863	0.865
GAN 2-hop Neighborhood	0.983	0.979	0.982	0.980
GAN 3-hop Neighborhood	0.995	0.991	0.995	0.993

Supplementary Table 1: Evaluation of different models to explore the impact of extended neighborhoods for the predictive power of NEPSTA GIN: Graph isomorphic network, GAN: Graph attention network. The model evaluation was performed on 84 patients.
